# Transient Quadriplegia With Albuminocytologic Dissociation: A Unique Presentation of Suspected Guillain-Barré Syndrome

**DOI:** 10.7759/cureus.86213

**Published:** 2025-06-17

**Authors:** Ryan Nazari, Jonathan C Hall

**Affiliations:** 1 College of Osteopathic Medicine, Kansas City University, Kansas City, USA; 2 Department of Internal Medicine, Overland Park Regional Medical Center, Overland Park, USA

**Keywords:** albuminocytologic dissociation (acd), guillan-barré syndrome (gbs), quadriplegia, transient quadriplegia, triplegia

## Abstract

Guillan-Barré syndrome (GBS) is an autoimmune-mediated process that typically causes a progressive ascending paralysis that can lead to persistence of symptoms, permanent paralysis, and even death. While most cases follow a classical course with gradual progression of symptoms until eventual plateau, atypical and rapid onset clinical presentations can complicate differential diagnosis and diagnostic certainty, leading to late diagnosis by clinicians. A 35-year-old male with a history of intravenous (IV) methamphetamine use presented with a sudden onset and complete motor and sensory triplegia of the bilateral legs and left arm upon waking, which resolved shortly after hospital admission. After transfer for neurological referral, his only complaint of distal extremity paresthesia improved daily until hospital day 3, in which he experienced acute quadriplegia with complete sensory and motor loss, prompting stroke protocol activation. Neuroimaging was unremarkable, but cerebrospinal fluid (CSF) analysis revealed elevated protein (87.3 mg/dL) with albuminocytologic dissociation (ACD). The patient was subsequently initiated on intravenous immunoglobulin (IVIg); however, his symptoms had almost completely resolved prior to the initiation of this treatment. He ultimately experienced full recovery without residual deficits. This case highlights a rare and diagnostically challenging presentation of suspected GBS, marked by transient and recurrent neurologic deficits rather than the classic monophasic presentation. Such presentations may be mistaken for functional disorders or overlooked entirely, delaying appropriate neurological evaluation. Despite rapid resolution of symptoms and other possible underlying etiologies in this case, hallmark CSF findings of elevated protein and ACD supported the diagnosis. This underscores the importance of maintaining a high index of suspicion for GBS in patients with unexplained neurological deficits, especially in the context of a recent illness. Early recognition remains critical to guide management and prevent severe complications.

## Introduction

Guillan-Barré syndrome (GBS) is the most common acute cause of flaccid paralysis worldwide, with several different etiologies having been noted, with particular emphasis and documentation of microorganisms as the offending agents [1]. In an estimated 100,000 new cases annually, the most common presentation includes an antecedent illness, with a progressive onset of limb weakness, hyporeflexia, paresthesias, and pain, often described as ascending paralysis, which can last up to four weeks before reaching a plateau [1-5]. Of the microorganisms associated with GBS, *Campylobacter jejuni *is the most notable, theorized to affect the peripheral nervous system through an autoantibody-mediated immune process using molecular mimicry; these patients typically experience more rapid deterioration [1-2,5]. Severe cases of GBS can include polyneuropathy that ascends to the level of the diaphragm, resulting in complete paralysis and respiratory failure, requiring mechanical intubation [1-3]. Severity of disease seems to be correlated with disease etiology and patient risk factors; nonetheless, even after treatment, many patients have been noted to experience persistent fatigue, with up to 20% unable to walk, and even death in 3-10% [3-4].

There have been several sets of criteria proposed regarding the diagnosis of GBS. Early recognition for clinicians is essential, given the risk of chronic sequelae and acute mortality in these patients. In 1976, the first diagnostic criteria for GBS were created, not to ensure early recognition of the disease process but to evaluate rates of GBS in association with vaccines given in the United States; these criteria were later reaffirmed and expanded upon in 1990 [5]. In 2011, the Brighton Collaboration, sponsored by the World Health Organization, sought to bring forth increased diagnostic certainty in GBS, with these definitions referred to as the “Brighton Criteria.” The Brighton Criteria grades findings with points based on the following clinical criteria: flaccid limb weakness, decreased/absent deep tendon reflexes, monophasic course, absence of alternate diagnosis, and cerebrospinal fluid (CSF) findings such as increased protein concentration and decreased cell count, with additional findings from nerve conduction studies [5]. There seems to be consensus among clinicians that the hallmark findings in these patients include increased CSF protein concentration with normal CSF white blood cell count, referred to as albuminocytologic dissociation (ACD), on analysis [1,3,5].

While the classical presentation and diagnostic framework for GBS are well-established, atypical or transient symptomatology can present diagnostic challenges, particularly when symptoms resolve rapidly or occur in unusual patterns. We present a rare case of suspected GBS in a patient with episodic, transient triplegia and quadriplegia with full recovery, emphasizing the importance of considering GBS even in non-classical presentations.

## Case presentation

A 35-year-old male with past medical history notable for intravenous (IV) methamphetamine use and prior gunshot wound to the right thigh presented to the hospital as a transfer from an off-site emergency department (ED) for neurological referral. The patient presented to the ED after waking up with paraplegia in the bilateral lower limbs and the left arm. The patient managed to call for help using his cellphone with his right arm and was subsequently transported via stretcher to an ambulance. His symptoms subsequently partially resolved in the ED, with the patient able to regain sensation to light touch in all affected extremities, with weak active movements, but was unable to walk. He also reported tingling sensations in all four extremities. The physicians began a workup for symptoms secondary to suspected conversion disorder and subsequently transferred the patient to our hospital for neurological evaluation.

On presentation to the hospital, the patient reported further alleviation of the weakness and numbness in the extremities, but with continued paresthesias in addition to fatigue. ROS was negative for headaches, chest pain, shortness of breath, nausea, vomiting, or changes in urination or bowel movements. Deep tendon reflexes were 1+ in both upper and lower extremities bilaterally. The patient did report pain in the left shoulder secondary to prior musculoskeletal injury, which was managed with acetaminophen and a lidocaine patch, as well as irritant contact dermatitis secondary to mechanical friction between the bilateral upper thighs, which was alleviated with dry powder application. The patient additionally reported a history of illness, which resolved one week prior to presentation, with a runny nose, nasal congestion, and a productive cough. The patient’s last known IV drug use was 48 hours prior to initial ED presentation.

**Figure 1:**
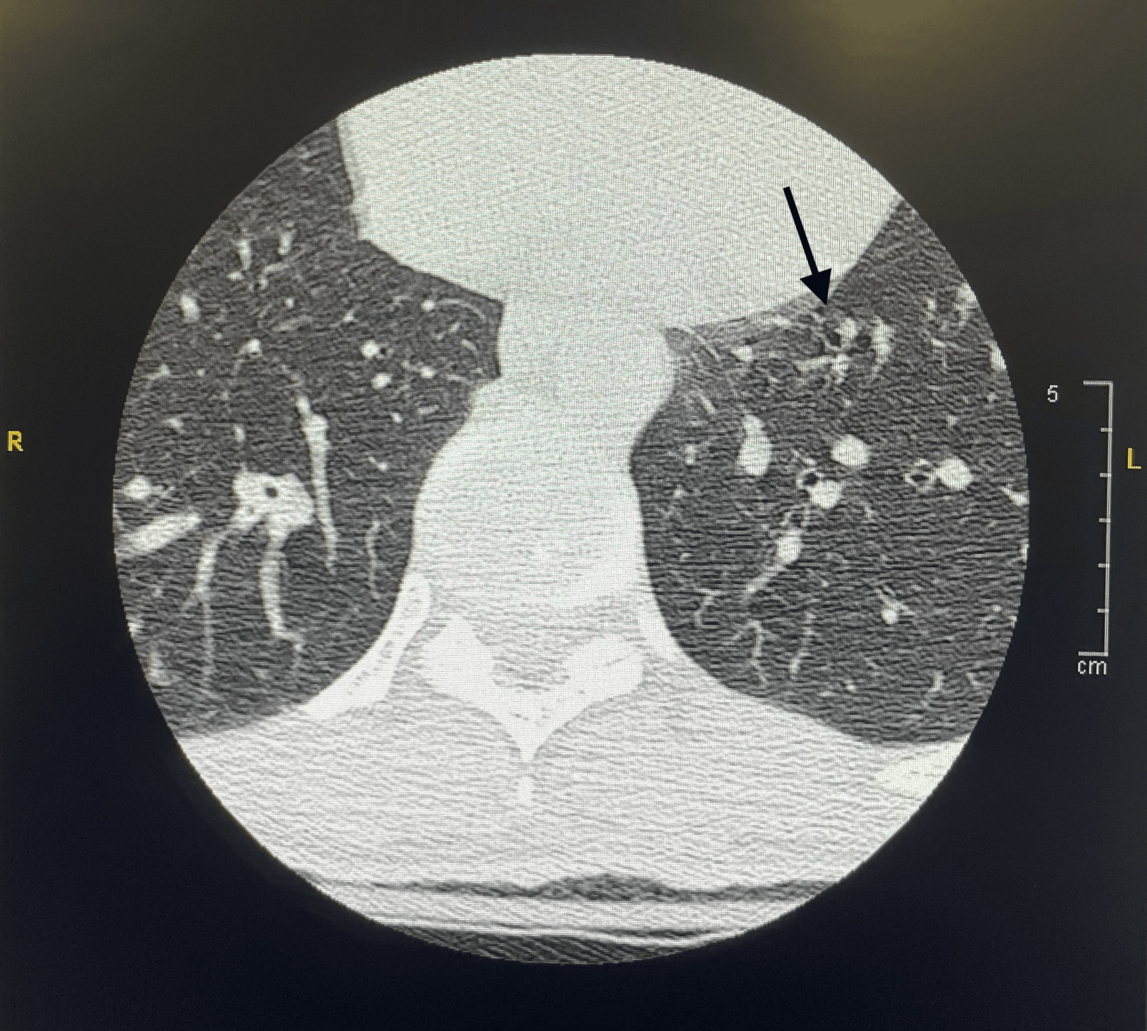
Axial chest CT image demonstrating left lower lobe consolidation (black arrow) with air bronchograms.

Patient imaging included brain, cervical, and lumbar MRI, with no acute findings other than mild spondylosis in the lumbar region. CT angiography of the head and neck region and bilateral lower extremity venous duplex ultrasonography were unremarkable. Chest CT (Figure 1) demonstrated left lower lobe consolidation with suspected aspiration pneumonia, which was managed with amoxicillin/clavulanate.

On day two of admission, the patient endorsed improvement in all symptoms, and the only remaining complaint was tingling sensations in the bilateral fingertips. On the night of day three, the patient experienced a sudden onset of quadriplegia, with complete motor and sensory loss, including to painful stimuli. A visitor of the patient reported that he had fallen asleep rather quickly compared to baseline, and within an hour began to breathe heavily in his sleep. Nursing staff were then alerted and assessed the patient, prompting stroke protocol activation and transfer to the Intensive Care Unit (ICU). The stroke work-up was unremarkable; however, a subsequent lumbar puncture revealed cerebrospinal fluid with an elevated protein level of 87.3 mg/dL and ACD on analysis, as well as an unremarkable white blood cell count, glucose level, and opening pressure. The patient was subsequently started on a five-day course of intravenous immunoglobulin (IVIg); however, by the time treatment was initiated, he was already demonstrating signs of recovery from his transient paraplegia, which had lasted only a few hours.

**Figure 2:**
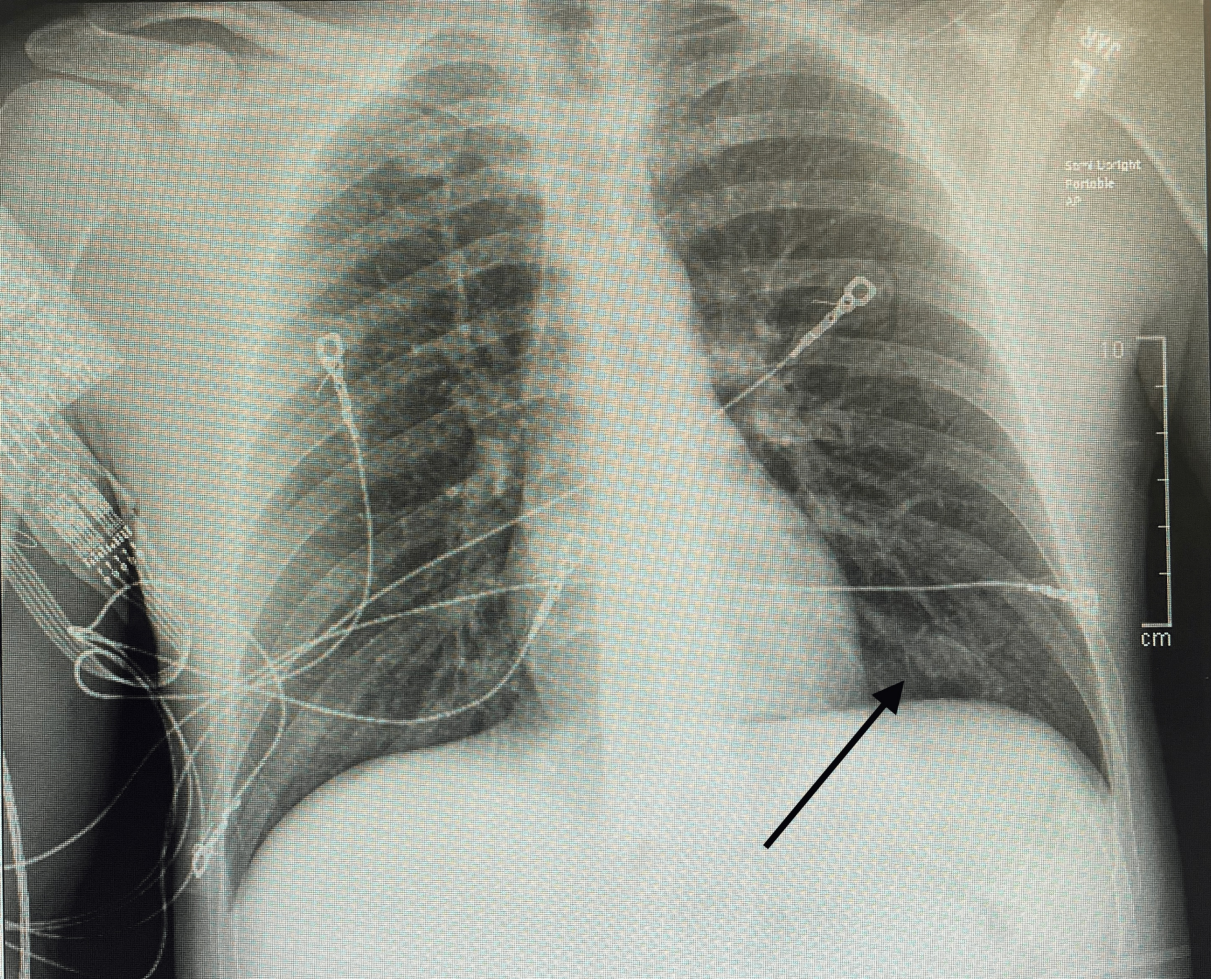
Portable anteroposterior chest radiograph demonstrating clear lung fields without evidence of consolidation, effusion, or pneumothorax. A black arrow highlights the representative normal left lower lobe region.

The remainder of the hospital course of this patient was unremarkable. He experienced daily improvement of symptoms, which were limited to left upper extremity paresthesia confined to the fingertips. The patient was stepped down from the ICU, and both IVIg and antibiotic treatment courses were completed. Chest X-ray (Figure 2) demonstrated resolution of pneumonia. The patient was discharged from the hospital with full return to baseline and no subsequent follow-up.

## Discussion

GBS symptomatology and patient presentation can vary widely, with numerous factors at play [3]; however, certain attributes of the condition, like progressive ascending paralysis, are a mainstay in what is commonly defined and taught as GBS presentation. With the exception of nerve conduction studies, which were not performed, our patient’s presentation fulfills most components of level 1 diagnostic certainty, the highest level according to the Brighton Criteria for GBS. The only major inconsistency is the absence of a classic monophasic course, given the patient’s transient and recurrent deficits. Our patient experienced two separate episodes of complete paralysis, once of three extremities and once of all four, three days apart, which seemed to have self-resolved on each occasion. The rates of GBS resolution without treatment are not well-documented in the current literature. Hahn (1998) notes the presence of spontaneous gradual resolution in some GBS patients, typically over weeks to months after plateau [6]. The patient in this case was treated with IVIg, after which there were no subsequent episodes of neurological deficits, as well as complete resolution of lingering distal extremity paresthesia. According to Wang et al., spontaneous recovery of GBS can occur both during the acute phase and in the long-term follow-up, with no significant difference in long-term prognosis between patients treated with IVIg and those who received only supportive care [7]. Plasma exchange therapy is an additional treatment modality in GBS patients. This treatment approach was not pursued for our patient due to the hospital's lack of necessary resources and capabilities. Currently, both IVIg and plasma exchange have been shown to be equally effective in improving disease outcome by accelerating recovery, but neither halts disease progression or alters the extent of nerve damage [1]. Due to the nature of the patient’s transient and self-limiting episodes, it remains uncertain whether a definitive benefit was derived from IVIg therapy. Notably, this therapy is not without risks, particularly thromboembolic complications, which should be carefully considered when determining its use in a patient [1,3]. It is, however, important to note that the carried risk is more significant with trials of a second dose, which have failed to show improved clinical outcomes [1,3].

Transient paraplegia is neither an expected nor documented presentation nor subtype component of GBS. The most common subtype of GBS, associated with the previously referenced typical symptoms, is defined as acute inflammatory demyelinating polyneuropathy (AIDP). Other subtypes can involve differences in organ system involvement, sensory and motor involvement, and specific extremity involvement. GBS groupings outside of AIDP rely less heavily on CSF findings and rather include increased emphasis on electrophysiological criteria for diagnosis [1,3-4]. Bourque et al. demonstrated a GBS diagnostic sensitivity of 70% in patients with total CSF protein greater than the upper limit of normal (0.45g/L) within zero to six days of initial symptom onset [8]. Interestingly, in a sample of 846 patients, Hakem et al. found a 70% GBS sensitivity in CSF examination positive for ACD, which increased to 84% in samples taken after four days of weakness onset [9]. Therefore, while ACD is a useful diagnostic marker, its absence does not exclude the diagnosis of GBS, especially in the early stages of the disease. Additionally, the lack of characteristic CSF findings alone is insufficient to rule out GBS, underscoring the importance of thorough evaluation when clinical suspicion remains high.

Transient or episodic paraplegia, as observed in our patient, is exceedingly rare and not well-documented in the literature. This presentation led to diagnostic uncertainty, particularly as the paralysis resolved spontaneously and rapidly. Atypical manifestations of GBS, such as recurrent or episodic weakness, may be misinterpreted as psychogenic or functional neurological disorders, particularly in patients with a history of substance use or psychiatric comorbidities [10]. A 2021 study highlighted that individuals with a history of drug use may have their health concerns overlooked and receive suboptimal treatment [11]. In our patient, initial evaluation may have displayed anchoring bias, in focusing on conversion disorder, given the transient nature of his symptoms and the history of IV methamphetamine use, delaying more definitive neurological work-up. Although the episodes of paralysis in this patient were transient, earlier identification and initiation of treatment may have prevented the second paralytic episode, as well as the stroke protocol activation that ensued. This rare and anomalous case highlights the need for clinicians to consider GBS in the differential diagnosis of transient or episodic neurological deficits, particularly when accompanied by findings such as ACD in CSF analysis, as well as consideration of repeat neurological assessments and CSF analysis to capture evolving diagnostic markers, to ensure timely, appropriate intervention.

## Conclusions

This case underscores the diagnostic challenges associated with an atypical presentation of suspected GBS characterized by transient, episodic motor and sensory tri- and paraplegia, which has yet to be documented in the literature. The case description seeks to increase clinical acumen in the possibility of this atypical presentation of GBS, to encourage providers to decrease the threshold for full and thorough neurological work-up in patients presenting with neurologic deficits, as well as provide a brief review of the diagnosis and management of GBS.
